# An initial assessment of an opinion leader-informed intervention to improve concussion-related outcomes among middle school parents: a randomized controlled trial

**DOI:** 10.1080/07853890.2024.2393760

**Published:** 2024-08-25

**Authors:** Zachary Yukio Kerr, Brittany M. Ingram, Samuel Livingston, Paula Gildner, Johna K. Register-Mihalik

**Affiliations:** a Department of Exercise and Sport Science, Injury Prevention Research Center, University of North Carolina at Chapel Hill, Chapel Hill, NC, USA; b Department of Exercise and Sport Science, Matthew Gfeller Center, Human Movement Science Curriculum, University of North Carolina at Chapel Hill, Chapel Hill, NC, USA; c Injury Prevention Research Center, The University of North Carolina at Chapel Hill, Chapel Hill, NC, USA; d Department of Exercise and Sport Science, STAR Heel Performance Laboratory, Matthew Gfeller Center and Injury Prevention Research Center, University of North Carolina at Chapel Hill, Chapel Hill, NC, USA

**Keywords:** Traumatic brain injury, youth sports, education, knowledge, attitudes, intention, self-efficacy

## Abstract

**Introduction:**

There is a need for evidence-based prevention programming that can reduce head impacts and increase reporting and disclosure of concussion. This study assessed an intervention to decrease concussion risk and improve concussion management through improving concussion-related knowledge, attitudes, intentions, and self-efficacy among parents in the middle school (MS) sport setting.

**Patients and methods:**

This randomized controlled trial (NCT04841473) examined parents of MS-aged children. Participants were randomized into one of two study arms: (1) CDC, which completed an education training module that compiled concussion education from the Centers for Disease Control and Prevention (CDC); and (2) TRAIN + CDC, which completed the CDC training module and an additional TRAIN educational module that provided strategies (originating from the Popular Opinion Leader framework) on communicating such information with one’s personal peer networks and children. Validated measures of concussion-related knowledge, attitudes, intentions, and self-efficacy were collected before completing the training modules and one week following completion. Linear mixed model analyses examined differences in outcomes between study arms.

**Results:**

Overall, 103 parents completed the training modules and had valid pre- and post-intervention data (TRAIN + CDC *n* = 49; CDC *n* = 54). Analyses found that the study arms did not differ in the change scores from pre- to post-intervention across concussion-related outcomes. However, scores from pre- to post-intervention improved across both study arms for knowledge metrics, such as ‘Concussions are less likely to happen when athletes play by the rules of the sport’ (*p* < 0.001), and self-efficacy metrics, such as feeling confident in one’s knowledge and recognition of concussion symptoms (*p* < 0.001 and *p* = 0.001, respectively).

**Conclusions:**

Although study arms did not differ in change scores from pre- to post-intervention, beneficial increases were nonetheless found across both knowledge and self-efficacy. Additional research is needed to further examine the beneficial manners in which concussion education can be best delivered and the most effective.

## Introduction

An estimated 1.1–1.9 million sport-related traumatic brain injuries occur annually in US youth under 18 years of age [1]. A common implementation strategy, primary prevention, is limiting the frequency and magnitude of head impact in youth sport [2]. Although some sports administrators and coaches have adopted such strategies to mitigate head contact (such as rule changes, or modified practice drills) [3–8], head contact is still prevalent in many youth sports. In addition, managing concussion is challenging due to the non-disclosure of concussion symptoms by youth athletes to healthcare providers, parents, or coaches; up to 50% of concussions in youth athletes are never reported to a health care provider [9], and this proportion may be even higher in settings with limited access to providers, such as middle schools, an intermediary level of education with students aged 10–15 years [10]. Thus, there is a pressing need for evidence-based prevention programming that can reduce head impacts and increase reporting and disclosure of concussion.

Currently, the Centers for Disease Control and Prevention (CDC) provide educational resources related to concussion prevention in youth athletes [11]. However, empirical studies observed mixed results for provisions of concussion education concerning improvements in knowledge, behaviors, and attitudes [12–16]. At the same time, concussion-related prevention programming in middle school sport settings needs to consider additional characteristics. For example, middle school sport settings may have limited interactions among coaches and parents unless directly related to logistics, such as scheduling [10]. Additionally, communication is often more prevalent when concussions and other injuries occurred on a team [10]. Also, previous research with parents of middle school children estimates one-third of parents did not access doctors/healthcare providers and other healthcare-related resources, such as the CDC for sport-related concussion information [17].

Given these concerns, an ideal concussion-related prevention program would help parents not only obtain education but also develop strategies to discuss such resources within their peer networks for wider uptake and dissemination within this setting to improve concussion prevention and management. Interventions in other public health fields, such as the Popular Opinion Leader (POL) intervention [18,19], discuss tenets of communication that can help the dissemination of information. These include the discussion of risk reduction strategies and the dispelling of associated myths and misconceptions with ‘I’ statements that emphasize personal endorsement [18]. As a result, this study aimed to examine concussion education training modules that compiled CDC-related concussion education information while providing strategies for communicating such information with parents’ personal peer networks and children. The randomized controlled trial (RCT) study design then determined the effect of concussion education training modules on concussion-related knowledge, attitude, intentions, and self-efficacy among parents in the middle school sports setting.

## Patients and methods

This RCT (NCT04841473) was designed with the goal of performing an initial assessment of a new POL-informed intervention to decrease concussion risk and improve concussion management through changes to concussion-related knowledge, attitudes, intentions, and self-efficacy among parents in the MS sport setting. The online platform surveyed parents of MS-aged children to assess the effects of the intervention. All online materials were hosted on Qualtrics (Qualtrics Inc., Provo, UT, USA). The study was approved by the Institutional Review Board at The University of North Carolina at Chapel Hill. All participants provided written informed consent before participating in the study. The study adhered to CONSORT guidelines (document can be found at https://dataverse.unc.edu/dataset.xhtml?persistentId=doi:10.15139/S3/2N0Y9J).

### Sampling and data collection

**Figure 1.**
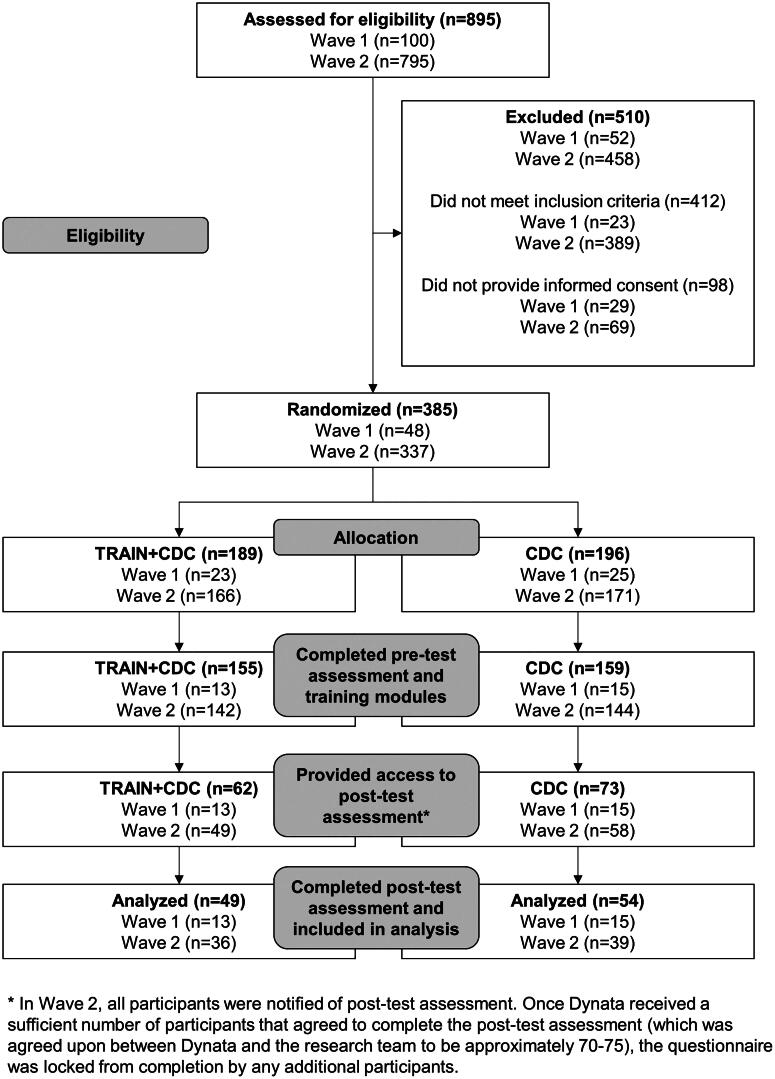
CONSORT diagram.

The population of interest is parents of MS-aged children residing in the US. Due to the COVID-19 pandemic, all recruitment was done virtually. Inclusion criteria for the study was the following: (1) US resident; (2) parent/guardian of a child enrolled in a MS; and (3) MS child had participated in organized sports in the past two years before recruitment (Figure 1). The study sample was recruited in two waves, with details included below. As a novel study in this area, no *a priori* sample size was calculated, although we originally aimed to obtain a sample of at least 180 parents. However, given changes to recruitment efforts due to constraints caused by the COVID-19 pandemic, this sample size goal dropped to 100.

#### Wave 1

Wave 1 occurred from May to August 2021. To recruit the sample, online flyers were posted on social media and informational emails were sent to organizations and agencies that work with youth (e.g. YMCAs, city/county parks and recreation, club sports leagues and organizations, and other community-based sports leagues). Online flyers and informational emails included a link to the online questionnaire. Those interested in participating in the study had to first complete an online screener to determine eligibility (see Figure 1 for information on recruitment numbers).

Upon obtaining informed consent from those who were eligible, simple random sampling was used to place individuals into one of two study arms: (1) the control group (CDC), who completed solely standard CDC concussion education training; and (2) the treatment group (TRAIN + CDC), who completed both the TRAIN education training and standard CDC concussion education training (see below for module information). After the randomization process, participants were provided with an online self-administered pre-test questionnaire (see below for information on outcome measures and covariates of interest). After completing the training modules, participants were notified they would be recontacted in seven days to complete a post-test questionnaire. The post-test questionnaire included items related to participants’ evaluations of the training modules and the outcomes of interest previously acquired in the pre-test questionnaire. Upon completion of the study, a gift card incentive was provided to participants.

#### Wave 2

Wave 2 occurred from September to October 2021, with the aim of increasing recruitment numbers. To recruit the sample, we worked with Dynata, which used a panel of US residents that had agreed to participate in online survey research. Dynata used certification processes, such as digital fingerprinting, IP-verification, and built-in quality control questions to ensure data quality. Individuals recruited into the Dynata participant pool had provided demographic information from which Dynata would identify those eligible for specific studies. For this study, Dynata identified those individuals in their participant pool with children aged 10–15 years. Among this group of participants, Dynata randomly generated a sample that received an invitation to participate in this study. To avoid self-selection bias, specific study details were not included in the invitation; instead, participants were invited to ‘take a survey’. Study details were then provided upon accepting the invitation. The beginning of the survey included the eligibility screener completed by participants in Wave 1 (see Figure 1 for recruitment numbers). It should be noted that Dynata purposely over-recruited, aware that it would lose potential participants due to not meeting inclusion criteria, not providing informed consent, and not completing the study.

Upon obtaining informed consent from those who were eligible, Dynata’s internal processes then automatically randomized individuals into one of the two study arms. Participants completed the pre-test questionnaire and then completed the relevant training module(s) associated with their study arm. After completing the training module(s), participants were notified they would be recontacted in seven days to complete a post-test questionnaire. Dynata reimbursed participants with ‘reward points’ that could be redeemed for cash, gift cards, or other incentives. Part of Dynata’s over-recruitment strategy aimed to ensure a sufficient number of post-test assessments could be obtained (given concerns about loss to follow-up). Once Dynata received a sufficient number of participants that agreed to complete the post-test assessment (which was agreed upon between Dynata and the research team to be ∼70–75), the questionnaire was locked from completion by additional participants.

### Development of training modules

Participants in the CDC study arm received CDC standard concussion education materials focused on concussion prevention in youth sport. These materials were acquired from CDC-generated resources but compiled together by study staff. The objectives of the CDC training module were to (1) provide general information on concussion; (2) identify common signs and symptoms of concussion; and (3) describe action steps if an MS child suffers a concussion.

In the TRAIN + CDC study arm, participants received the same education materials as the CDC study arm along with a second training module (TRAIN), which focused on providing more in-depth concussion information related to reducing the number of concussions through safer sports play and responding appropriately when a concussion occurs. The TRAIN module also aimed to help participants identify ways to share up-to-date concussion related information. This latter aim was constructed around the tenets of a POL intervention [18,19], which focused on the importance of discussing with peers the importance of the awareness and prevention of the outcome of interest (i.e. concussion), correcting associated myths and misconceptions, and describing strategies to reduce risk using ‘I’ statements that emphasize personal endorsement.

Before the study, both training modules underwent refinement through consultations with experts in sports medicine, athletic training, and public health. As the POL intervention was originally developed to reduce risk related to the Human Immunodeficiency Virus (HIV) [19], we also sought feedback from experts that had previously used it in their frontline HIV prevention work. We also piloted the modules with 20 parents of MS athletes and obtained feedback *via* cognitive interviews. Specific feedback was provided concerning content, delivery, accessibility, and overall impact. Feedback was then reviewed and categorized by the research team to allow for adaptation of the training modules to better fit the MS parent population.

### Measures

#### Exposures

The main exposure of interest was the study arm into which participants were randomized: (1) CDC, which completed solely standard CDC concussion education training; and (2) TRAIN + CDC, which completed the standard CDC concussion education training as in the control group and the additional TRAIN educational module.

#### Outcomes

Outcomes of interest originated from questionnaire data. The post-test questionnaire included measures focusing on participants’ evaluation of the training modules (see Table 2 for the specific items). This included: an overall evaluation on a 1–10 scale, with 10 representing the most positive evaluation; and additional specific measures on scales from 1 to 5. The first set of these specific measures focused on how helpful (1 = Not helpful at all, and 5 = Very helpful) specific information was from the training modules. The second set of specific measures focused on how participants agreed (1 = Strongly disagree, and 5 = Strongly agree) with statements regarding the value of the content and their willingness to recommend the modules to others.

**Table 2.** Evaluation of training modules by participants (*n* = 103).

Evaluation metric	Study arm		Comparison *p*-value^a^
	TRAIN + CDC (*n* = 49)	CDC (*n* = 54)	
Overall evaluation [Mean (*SD*)]^b^	8.9 (1.4)	8.7 (1.5)	0.60
How helpful was the following information from the training?^c^			
General concussion information	4.5 (0.8)	4.6 (0.8)	0.54
Reducing concussion risk	4.3 (0.8)	4.4 (0.8)	0.53
Responding to concussions	4.5 (0.8)	4.5 (0.7)	0.89
Keys to effective conversations	4.2 (0.8)	4.3 (0.9)	0.41
Rate how much you agree or disagree with the following statements^d^			
The content of the training was informative.	4.6 (0.7)	4.7 (0.5)	0.34
I will use the information I learned from the training on a regular basis.	4.2 (0.7)	4.4 (0.7)	0.29
The time commitment for the training modules was appropriate.^e^	4.2 (0.9)	4.6 (0.6)	0.02*
I would recommend the modules to other parents.	4.4 (0.7)	4.6 (0.6)	0.13

*Represents *p*-value <0.05.

^a^Group comparisons conducted with independent sample *t*-tests.

^b^Participants provided feedback on a 1–10 scale, with 10 representing the highest evaluation.

^c^Participants provided feedback on a 1–5 scale, with 1 = Not helpful at all, and 5 = Very helpful.

^d^Participants provided feedback on a 1–5 scale, with 1 = Strongly disagree, and 5 = Strongly agree.

^e^The median time for the TRAIN + CDC study arm to complete their training module was 55.6 min (Interquartile range of 40.8–83.9 min). The median time for the CDC study arm to complete their training module was 36.6 min (Interquartile range of 21.9–48.5 min).

The pre-test and post-test questionnaires included measures related to concussion-related knowledge, attitude, intentions, and self-efficacy (see Tables 3–5 for the specific items). Measures were based on previously developed and refined measurements [20–24].

**Table 3.** Concussion-related knowledge outcome comparisons by group (*n* = 103).

Outcome items^a^ [Mean (*SD*)]	Study arm				*p*-values from linear mixed model		
	TRAIN + CDC (*n* = 49)		CDC (*n* = 54)				
	Pre	Post	Pre	Post	Group	Time	Group*time
Please indicate how strongly you agree or disagree with the following statements							
Concussions are less likely to happen when athletes play by the rules of the sport.	2.9 (0.9)	3.3 (0.7)	3.0 (1.0)	3.4 (0.7)	0.31	<0.001*	0.99
Concussions are less likely to happen when athletes reduce head contact.	3.3 (0.7)	3.5 (0.6)	3.5 (0.6)	3.5 (0.6)	0.32	0.17	0.25
Concussion symptoms typically go away sooner when a youth athlete is taken out of a game/practice when they are suspected of having a concussion.	3.0 (0.9)	3.2 (0.9)	3.0 (1.0)	3.5 (0.7)	0.25	0.002*	0.12
As long as symptoms resolve within 15 min, a youth athlete who exhibits signs and symptoms consistent with a concussion can be returned to play on the same day.	2.0 (1.1)	2.0 (1.2)	2.2 (1.2)	2.3 (1.2)	0.16	0.67	0.67
A youth athlete exhibiting signs and symptoms consistent with a concussion should complete a return to play protocol before returning to sport.	3.4 (1.0)	3.5 (0.9)	3.5 (0.8)	3.4 (0.9)	0.76	0.74	0.38

*Represents *p*-value <0.05.

^a^For each item, participants provided feedback on a 1–4 scale, with 1 = Strongly disagree, and 4 = Strongly agree. The low Cronbach’s alpha (*α* = 0.58) resulted in keeping analyses for each item discrete.

**Table 4.** Concussion-related attitude outcome comparisons by group (*n* = 103).

Outcome scales^a^ [Mean (*SD*)]	Study arm				*p*-Values from linear mixed model		
	TRAIN + CDC (*n* = 49)		CDC (*n* = 54)				
	Pre	Post	Pre	Post	Group	Time	Group*time
Talking to my youth athlete who plays sports about playing by the rules of their sport is…	32.7 (3.6)	32.0 (4.7)	32.9 (3.8)	33.1 (3.6)	0.31	0.51	0.20
Talking to my youth athlete who plays sports about reducing contact is…	30.3 (5.3)	31.4 (4.8)	31.2 (4.8)	31.9 (5.7)	0.84	0.43	0.36
Taking my youth athlete out of a game/practice when they may have a concussion is…	31.4 (5.5)	32.1 (4.6)	32.2 (4.5)	32.8 (4.2)	0.33	0.26	0.93
Seeking immediate medical care for my youth athlete when they may have a concussion is…	32.3 (4.1)	32.3 (4.5)	32.9 (3.4)	32.8 (4.5)	0.43	0.89	0.89

*Represents *p*-value <0.05.

^a^For each scale, participants provided feedback on a 1–7 scale on five components: irresponsible…responsible; harmful…beneficial; extremely difficulty…extremely easy; bad…good; and unimportant…important. Higher values for each item equated to more favorable attitudes (e.g. 1 = Irresponsible, and 7 = Responsible). Scale measure was the sum of each item (potential range of 5–35). Cronbach’s alphas for each scale were 0.83, 0.92, 0.87, 0.87, respectively).

**Table 5.** Concussion-related intention and self-efficacy outcome comparisons by group (*n* = 103).

Please indicate how strongly you agree or disagree with the following statements^a^ [Mean (*SD*)]	Study arm				*p*-Values from linear mixed model		
	TRAIN + CDC (*n* = 49)		CDC (*n* = 54)				
	Pre	Post	Pre	Post	Group	Time	Group*time
INTENTION I plan to…							
1. …take my child out of a game/practice when they may have a concussion.	3.7 (0.7)	3.8 (0.5)	3.8 (0.5)	3.9 (0.4)	0.39	0.19	0.70
2. …seek immediate medical care for my child when they may have a concussion.	3.7 (0.5)	3.7 (0.6)	3.7 (0.5)	3.7 (0.5)	0.84	0.88	0.59
3. …talk to others (e.g. my child, other parents, etc.) about playing by the rules of the game.	3.4 (0.6)	3.6 (0.6)	3.5 (0.7)	3.7 (0.5)	0.27	0.02*	0.43
4. …talk to others (e.g. my child, other parents, etc.) about reducing head contact.	3.5 (0.6)	3.5 (0.6)	3.5 (0.7)	3.6 (0.6)	0.49	0.24	0.37
SELF-EFFICACY I feel confident…							
1. …in my knowledge of concussion symptoms.	3.3 (0.8)	3.6 (0.6)	3.3 (0.7)	3.6 (0.6)	0.97	<0.001*	0.95
2. …in my ability to recognize concussion symptoms.	3.2 (0.8)	3.5 (0.5)	3.3 (0.7)	3.6 (0.5)	0.61	0.001*	0.97
3. …taking my child out of the game/practice when they may have a concussion.	3.6 (0.7)	3.7 (0.5)	3.7 (0.5)	3.8 (0.5)	0.66	0.16	0.84
4. …seeking immediate medical care for my child when they may have a concussion.	3.6 (0.6)	3.6 (0.6)	3.5 (0.7)	3.7 (0.5)	0.97	0.11	0.38
I feel confident talking to…							
1. …my child/children about playing by the rules in youth sports.	3.8 (0.5)	3.7 (0.5)	3.8 (0.5)	3.7 (0.6)	0.96	0.17	0.69
2. …my child/children about reducing head contact in youth sports.	3.7 (0.5)	3.7 (0.5)	3.6 (0.6)	3.7 (0.5)	0.79	0.42	0.64
3. …other adults (e.g. parents, coaches, etc…) about playing by the rules in youth sports.	3.4 (0.6)	3.5 (0.7)	3.6 (0.7)	3.7 (0.6)	0.15	0.08	0.74
4. …other adults (e.g. parents coaches, etc…) about reducing head contact in youth sports.	3.4 (0.6)	3.4 (0.6)	3.4 (0.7)	3.5 (0.7)	0.82	0.18	0.70
5. …other adults (e.g. parents, coaches, etc…) about taking our children out of the games/practices when they may have a concussion.	3.6 (0.6)	3.5 (0.6)	3.6 (0.6)	3.6 (0.6)	0.44	0.77	0.32
6. …other adults (e.g. parents, coaches, etc…) about seeking immediate medical care for our children when they may have a concussion.	3.4 (0.7)	3.5 (0.7)	3.7 (0.5)	3.6 (0.6)	0.05	0.41	0.09

*Represents *p*-value <0.05.

^a^For each item, participants provided feedback on a 1–4 scale, with 1 = Strongly disagree, and 4 = Strongly agree. The low Cronbach’s alphas for intentions (*α* = 0.56) and the two sets of self-efficacy measures (*α* = 0.63 and 0.78, respectively) resulted in keeping analyses for each item discrete.

Knowledge (Table 3) included five items related to the incidence, management, and recovery of concussion. For each item, participants provided feedback on a 1–4 scale, with 1 = Strongly disagree, and 4 = Strongly agree.

Attitudes (Table 4) focused on parent perceptions regarding four concepts: talking with their children about (1) playing by the rules, (2) reducing contact during sports participation; and when suspecting their child has a concussion, (3) taking their child out of sports participation, and (4) seeking immediate medical care. Each concept was measured in the context of five items on seven-point Likert-type scales: irresponsible…responsible; harmful…beneficial; extremely difficulty…extremely easy; bad…good; and unimportant…important. Higher values for each item equated to more favorable attitudes (e.g. 1 = Irresponsible, and 7 = Responsible). Higher scores indicated more favorable attitudes.

Intentions (Table 5) included four items focused on parents’ plans to engage in activities related to concussion management and discuss safe gameplay with others. Self-efficacy included: first, four items on parents’ confidence in their concussion knowledge and ability to manage suspected concussions; and second, six items on confidence in talking about safer gameplay with others. For each item related to intentions and self-efficacy, participants provided feedback on a 1–4 scale, with 1 = Strongly disagree, and 4 = Strongly agree.

#### Covariates

The pre-test questionnaire acquired measures for age (in years), gender, race/ethnicity, highest education level, whether the participant or their MS child had a concussion history and previous formal concussion education. A previously-validated competitiveness index [25] was also included, which utilized 14 5-point scale items focused on ‘interpersonal competitiveness in every context’. Resulting scores ranged from 5 to 70 (*α* = 0.84); higher scores indicated a more competitive nature. Participants were also asked about the sports that their MS-aged children had previously played, from a set of 14 sports, with an additional ‘fill-in other’ option.

### Statistical analysis

Data were analyzed using SAS (Version 9.4; SAS Institute Inc., Cary, NC, USA). All analyses used an alpha of 0.05. Descriptive analyses, including frequencies and measures of central tendency and variability, were conducted for all measures of interest. For outcome scale measures (i.e. knowledge, attitudes, intentions, self-efficacy), internal consistency was assessed with Cronbach’s alphas; low Cronbach’s alpha (*α* < 0.80) resulted in keeping analyses for each item discrete as opposed to using the scale measures as intended.

Comparative analyses (e.g. independent sample t-tests, chi-square tests) examined differences of characteristics between the study arms. For race/ethnicity, categories assessed were ‘White/non-Hispanic’ and ‘Other race/ethnicity’; for highest education level, ‘Less than bachelor’s degree’ and ‘Bachelor’s degree or higher’; and for sport level previously played by MS child, ‘contact’, ‘limited contact’, and ‘non-contact’. Contact level classifications originate from Rice et al. [26] If children played multiple sports, they were included in each sport-specific category. However, for contact level, they were categorized into the highest contact level group (e.g. a child participating in ice hockey and tennis was classified in the ‘contact sports’ category). Any variables with differences detected were to be included in subsequent analyses as covariates.

Next, scores for each evaluation measure were compared between study arms with independent sample t-tests. Last, linear mixed model analyses with random intercepts were used to examine differences in outcomes between study arms. The research team discussed how to examine covariates within models. We had identified gender and race/ethnicity as key covariates of interest [22,27–29] that were best treated as effect measure modifiers. Given sample size concerns for those identifying as ‘Other race/ethnicity’, only gender-specific analyses were conducted with all linear mixed model analyses. Effect sizes (Cohen’s *d*) with 95% confidence intervals (CI) were provided for statistically significant findings in the linear mixed model analyses [30]. Effect sizes were deemed as small (0.20 < *d* < 0.49), medium (0.50 < *d* < 0.79), and large (*d* ≥ 0.80) [31]. An *a priori* decision was made to examine the scale measures with parametric methods given previous findings [32,33].

## Results

### Participant descriptives

Overall, 103 individuals completed their allocated training modules and the pre- and post-tests and had valid data (28 from Wave 1, 75 from Wave 2). Of these, 49 individuals had been allocated to the TRAIN + CDC study arm, and 54 individuals had been allocated to the CDC control study arm.

Most participants identified as female (TRAIN + CDC: 51.0%; CDC: 55.6%) and White/non-Hispanic (TRAIN + CDC: 77.6%; CDC: 77.8%), with a bachelor’s degree or higher (TRAIN + CDC: 89.8%; CDC: 88.9%; Table 1). About one-third had previously sustained a concussion (TRAIN + CDC: 30.6%; CDC: 33.3%) and over half had previous formal concussion education (TRAIN + CDC: 57.1%; CDC: 61.1%). Participants reported that their MS-aged children played a variety of sports with the most reported being basketball and soccer. No participant differences were found between study arms. No differences in participant characteristics were found (Table 1).

**Table 1.** Characteristics of participants (*n* = 103).

Characteristic	Study arm		Comparison *p*-value^a^
	TRAIN + CDC (*n* = 49)	CDC (*n* = 54)	
Age [Mean (*SD*)]	42.7 (8.6)	43.0 (7.6)	0.38
Gender [*n* (%)]			0.65^b^
Female	25 (51.0)	30 (55.6)	
Male	24 (49.0)	24 (44.4)	
Other gender identity	0	0	
Race/ethnicity [*n* (%)]			0.98^c^
White/non-Hispanic	38 (77.6)	42 (77.8)	
Other race/ethnicity	11 (22.4)	12 (22.2)	
Black/Hispanic	1 (2.0)	1 (1.9)	
White/Hispanic	3 (6.0)	6 (11.1)	
Asian/non-Hispanic	2 (4.1)	2 (3.7)	
Black/non-Hispanic	4 (8.2)	2 (3.7)	
Other	1 (2.0)	1 (1.9)	
Highest education level [*n* (%)]			0.88^d^
Less than bachelor’s degree	5 (10.2)	6 (11.1)	
High school or GED	0	2 (3.7)	
Some college, no degree	1 (2.0)	1 (1.9)	
Associate degree	4 (8.2)	3 (5.6)	
Bachelor’s degree or higher	44 (89.8)	48 (88.9)	
Bachelor’s degree	24 (49.0)	26 (48.2)	
Master’s degree	15 (30.6)	17 (31.5)	
Professional degree	0	1 (1.9)	
Doctorate	5 (10.2)	4 (7.4)	
Personal concussion history [*n* (%)]			0.77
Yes	15 (30.6)	18 (33.3)	
No	34 (69.4)	36 (66.7)	
MS Child’s concussion history [*n* (%)]			0.15
Yes	8 (16.3)	15 (28.3)	
No	41 (83.7)	38 (71.7)	
Missing	0	1	
Previous formal concussion education [*n* (%)]			0.68
Yes	28 (57.1)	33 (61.1)	
No	21 (42.9)	21 (38.9)	
Competitiveness index [Mean (*SD*)]	47.7 (8.2)	44.9 (9.6)	0.12
Sport level previously played by MS child [*n* (%)]^e^			0.41^f^
Contact	43 (87.8)	40 (76.9)	
Basketball	31 (63.3)	29 (55.8)	
Soccer	27 (55.1)	17 (32.7)	
Tackle Football	12 (24.5)	7 (13.5)	
Cheer	6 (12.2)	9 (17.3)	
Lacrosse	5 (10.2)	3 (5.8)	
Field Hockey	5 (10.2)	4 (7.7)	
Diving	2 (4.1)	0 (0)	
Wrestling	2 (4.1)	4 (7.7)	
Ultimate Frisbee	1 (2)	1 (1.9)	
Ice Hockey	0 (0)	1 (1.9)	
Gymnastics	0 (0)	2 (3.8)	
Limited contact	3 (6.1)	7 (13.5)	
Baseball	19 (38.8)	21 (40.4)	
Volleyball	10 (20.4)	12 (23.1)	
Flag Football	7 (14.3)	10 (19.2)	
Softball	7 (14.3)	5 (9.6)	
Martial Arts	1 (2)	2 (3.8)	
Non-contact	3 (6.1)	5 (9.6)	
Tennis	12 (24.5)	14 (26.9)	
Cross Country	12 (24.5)	11 (21.2)	
Track and Field	7 (14.3)	6 (11.5)	
Jump Rope	2 (4.1)	2 (3.8)	
Golf	2 (4.1)	0 (0)	
Swimming	1 (2)	3 (5.8)	
Dance	1 (2)	1 (1.9)	
Archery	0 (0)	1 (1.9)	
Missing	0	2	

^a^Group comparisons conducted with independent sample *t*-tests for quantitative variables and chi-square tests for categorical variables, with the exception of sport played by MS child, which used a Fisher’s Exact Test.

^b^Group comparisons examined distributions between ‘Male’ and ‘Female’ only as all participants self-identified with these two categories.

^c^Group comparisons examined distributions between ‘White/non-Hispanic’ and ‘Other race/ethnicity’.

^d^Group comparisons examined distributions between ‘Less than bachelor’s degree’ and ‘Bachelor’s degree or higher’.

^e^Contact level classifications originate from Rice et al.^26^ If children played multiple sports, they were included in each sport-specific category. However, for contact level, they were categorized into the highest contact level group (e.g. a child participating in ice hockey and tennis was classified in the ‘contact sports’ category).

^f^Group comparisons examined distributions among ‘Contact’, ‘Limited contact’, and ‘Non-contact’ categories.

Among the 49 participants randomized to the TRAIN + CDC study arm, the median time to completion was 55.6 min (Interquartile range of 40.8–83.9 min). Among the 54 participants randomized to the CDC study arm, the median time to completion was 36.6 min (Interquartile range of 21.9–48.5 min).

### Evaluation of training modules

Both study arms provided high overall evaluations of the training modules (Table 2). On a 1–10 scale, with 10 representing the most positive evaluation, the mean (*SD*) scores for the TRAIN + CDC and CDC study arms were 8.9 (1.4) and 8.7 (1.5), respectively. On 1–5 scale, with 5 representing the most positive evaluation, participants noted the modules being helpful with general concussion information [TRAIN + CDC: 4.5 (0.8); CDC: 4.6 (0.8)], responding to concussions [TRAIN + CDC: 4.5 (0.8); CDC: 4.5 (0.7)], and reducing concussion risk [TRAIN + CDC: 4.3 (0.8); CDC: 4.4 (0.8)]. On a 1–5 scale, with 1 representing ‘strongly disagree’ and 5 representing ‘strongly agree’, participants also noted that they would recommend the modules to other parents [TRAIN + CDC: 4.4 (0.7); CDC: 4.6 (0.6)]. The one significant difference found between study arms was regarding whether the time commitment was appropriate; on average, the CDC study arm found the time commitment of the training modules to be more appropriate than the TRAIN + CDC study arm [4.6 (0.6) *vs.* 4.2 (0.9); *p* = 0.02].

### Internal consistency in outcome scale measures

The low Cronbach’s alpha for concussion-related knowledge (*α* = 0.58) resulted in keeping analyses for each item discrete. Cronbach’s alphas (*α* = 0.83, 0.92, 0.87, 0.87, respectively) for the attitude-related measures led to using the scale measures as intended. The low Cronbach’s alphas for intentions (*α* = 0.56) and the two sets of self-efficacy measures (*α* = 0.63 and 0.78, respectively) resulted in keeping analyses for each item discrete.

### Comparisons of outcomes between study arms

Resultant analyses found that the study arms did not differ in the change in scores from pre- to post-intervention related to all concussion-related outcomes (Tables 3–5). However, time-based improvements in scores from pre- to post-intervention were seen across both study arms on a few outcomes. First, in relation to knowledge, increases across time were found for ‘Concussions are less likely to happen when athletes play by the rules of the sport’ [*p* < 0.001, *d* (95%CI) = 0.44 (0.23, 0.65)] and ‘Concussion symptoms typically go away sooner when a youth athlete is taken out of a game/practice when they are suspected of having a concussion’ [*p* = 0.002; *d* (95%CI) = 0.31 (0.08, 0.55); Table 3]. Second, in relation to intentions, increases across time were found for planning to talk to others (e.g. my child, other parents, etc.) about playing by the rules of the game [*p* = 0.02; *d* (95%CI) = 0.23 (0.03, 0.44); Table 5]. Last, in relation to self-efficacy, increases across time were found for feeling confident in the knowledge of concussion symptoms [*p* < 0.001; *d* (95%CI) = 0.39 (0.16, 0.62)], and in one’s ability to recognize concussion symptoms. [*p* = 0.001; *d* (95%CI) = 0.32 (0.07, 0.57)].

Analyses were then conducted stratified by gender, with fewer time-based significant findings found in those identifying as male *vs.* female (Table 6). Among those identifying as female, two group*time interactions were found. First, the CDC study arm had an increase in score from pre to post for ‘Concussion symptoms typically go away sooner when a youth athlete is taken out of a game/practice when they are suspected of having a concussion’ (2.9–3.4) whereas the TRAIN + CDC study arm had a slight decrease [3.2–3.0; interaction *p* = 0.009; *d* (95%CI) = −0.801 (−1.36, −0.24)]. Second, the CDC study arm had minimal change in score from pre to post for feeling confident talking to other adults (e.g. parents, coaches, etc.) about seeking immediate medical care for our children when they may have a concussion (3.7 to 3.7) whereas the TRAIN + CDC study arm had an increase [3.2–3.6; interaction *p* = 0.03; *d* (95%CI) = 0.49 (−0.06, 1.04)].

**Table 6.** Concussion-related outcome comparisons by group, stratified by gender.

Outcome measures [Mean (*SD*)]	Study arm among MALES				*p*-Values from linear mixed model		
	TRAIN + CDC (*n* = 24)		CDC (*n* = 24)				
	Pre	Post	Pre	Post	Group	Time	Group*time
KNOWLEDGE Please indicate how strongly you agree or disagree with the following statements^a^							
1. Concussions are less likely to happen when athletes play by the rules of the sport.	3.0 (1.0)	3.4 (0.6)	3.5 (0.6)	3.6 (0.6)	0.06	0.02*	0.17
3. Concussion symptoms typically go away sooner when a youth athlete is taken out of a game/practice when they are suspected of having a concussion.	2.8 (1.0)	3.4 (0.8)	3.1 (0.9)	3.7 (0.6)	0.11	<0.001*	0.90
Outcome measures [Mean (*SD*)]	Study arm among FEMALES				*p*-Values from linear mixed model		
	TRAIN + CDC (*n* = 25)		CDC (*n* = 30)				
	Pre	Post	Pre	Post	Group	Time	Group*time
KNOWLEDGE Please indicate how strongly you agree or disagree with the following statements^a^							
1. Concussions are less likely to happen when athletes play by the rules of the sport.	2.8 (0.8)	3.2 (0.9)	2.7 (1.1)	3.3 (0.7)	>0.99	<0.001*	0.25
3. Concussion symptoms typically go away sooner when a youth athlete is taken out of a game/practice when they are suspected of having a concussion.	3.2 (0.8)	3.0 (0.9)	2.9 (1.0)	3.4 (0.7)	0.88	0.44	0.009*
ATTITUDE Talking to my youth athlete who plays sports about reducing contact is…^b^	29.8 (5.7)	31.4 (4.2)	31.0 (5.4)	32.6 (4.5)	0.32	0.01*	0.91
INTENTION^c^ I plan to…							
3. …talk to others (e.g. my child, other parents, etc.) about playing by the rules of the game.	3.4 (0.6)	3.5 (0.6)	3.4 (0.7)	3.7 (0.6)	0.54	0.02*	0.54
4. …talk to others (e.g. my child, other parents, etc.) about reducing head contact.	3.3 (0.6)	3.5 (0.7)	3.4 (0.8)	3.7 (0.7)	0.50	0.01*	0.70
SELF-EFFICACY^c^ I feel confident…							
1 …in my knowledge of concussion symptoms.	3.0 (0.9)	3.6 (0.6)	3.1 (0.7)	3.7 (0.6)	0.78	<0.001*	0.85
2 …in my ability to recognize concussion symptoms.	3.0 (0.8)	3.5 (0.5)	3.2 (0.7)	3.6 (0.5)	0.31	<0.001*	0.84
4 …seeking immediate medical care for my child when they may have a concussion.	3.5 (0.7)	3.7 (0.5)	3.6 (0.8)	3.8 (0.5)	0.39	0.006*	0.80
I feel confident talking to…							
2. …my child/children about reducing head contact in youth sports.	3.6 (0.5)	3.7 (0.5)	3.6 (0.6)	3.8 (0.4)	0.43	0.04*	0.37
6. …other adults (e.g. parents, coaches, etc…) about seeking immediate medical care for our children when they may have a concussion.	3.2 (0.8)	3.6 (0.7)	3.7 (0.5)	3.7 (0.7)	0.053	0.03*	0.03*

*Notes:* Only outcomes with statistically significant findings (*p*-value <0.05) were included. All other outcomes had non-significant findings.

*Represents *p*-value <0.05.

^a^For each item, participants provided feedback on a 1–4 scale, with 1 = Strongly disagree, and 4 = Strongly agree.

^b^Participants provided feedback on a 1–7 scale on five components: irresponsible…responsible; harmful…beneficial; extremely difficulty…extremely easy; bad…good; and unimportant…important. Higher values for each item equated to more favorable attitudes (e.g. 1 = Irresponsible, and 7 = Responsible). Scale measure was the sum of each item (potential range of 5–35).

^c^For each item, participants provided feedback on a 1–4 scale, with 1 = Strongly disagree, and 4 = Strongly agree.

## Discussion

The recent efforts to develop strategies that ultimately aim to increase concussion prevention and management have focused on invested constituents, such as athletes, coaches, and parents [3–8]. Overall, there has been mixed findings, but recent research has highlighted the potential of programming that aims to invest in changing the norms associated with sport safety and culture [12–16]. However, research across the past decade has also noted difficulties in the implementation of concussion prevention programming; pertinent reasons included materials not being presented in easy-to-understand manners, insufficient time to participate in programming, and lack of buy-in [10,12,34].

Our study performed an initial assessment of a completely online training module aimed to help parents feel confident in discussing such prevention and management needs within their peer networks. The tenets of this training module were based on the POL intervention, which has been found to be effective within numerous populations and outcomes of interest [18,19]. Although both study arms received a training module focused on concussion-related education developed from CDC materials, the treatment study arm was also given this additional training module (TRAIN + CDC). Both modules were well received by research participants, with some evidence of increased knowledge a week after completing them. However, there was limited evidence that such increases differed between the study arms (see Table 7 for a summary of findings). Nonetheless, the findings highlight areas of future exploration that may help to improve the design and implementation of such training modules and that some key areas can be improved by interactive education with or without the POL components.

**Table 7.** Summary of key findings.

Outcome measures	Total sample (*n* = 103)	Males only (*n* = 48)	Females only (*n* = 55)	
	Time	Time	Time	Group*Time
KNOWLEDGE (Higher score = higher knowledge)				
1. Concussions are less likely to happen when athletes play by the rules of the sport.	Post > Pre	Post > Pre	Post > Pre	
3. Concussion symptoms typically go away sooner when a youth athlete is taken out of a game/practice when they are suspected of having a concussion.	Post > Pre	Post > Pre		Decrease in TRAIN + CDC study arm; Increase in CDC study arm
ATTITUDE (Higher score = more favorable attitudes)				
Talking to my youth athlete who plays sports about reducing contact is…			Post > Pre	
INTENTION (I plan to…) (Higher score = higher agreement)				
3. …talk to others (e.g. my child, other parents, etc.) about playing by the rules of the game.	Post > Pre		Post > Pre	
4. …talk to others (e.g. my child, other parents, etc.) about reducing head contact.			Post > Pre	
SELF-EFFICACY (Higher score = higher agreement)				
I feel confident…				
1. …in my knowledge of concussion symptoms.	Post > Pre		Post > Pre	
2. …in my ability to recognize concussion symptoms.	Post > Pre		Post > Pre	
4. …seeking immediate medical care for my child when they may have a concussion.			Post > Pre	
I feel confident talking to…				
2. …my child/children about reducing head contact in youth sports.			Post > Pre	
6. …other adults (e.g. parents, coaches, etc…) about seeking immediate medical care for our children when they may have a concussion.				Increase in TRAIN + CDC study arm; no change in CDC study arm

### Evaluation of the training modules

Both the CDC and TRAIN training modules were evaluated positively. The sole significant difference pertained to participants’ observations on the time required to complete the modules, which was foreseen as the TRAIN + CDC study arm was required to complete both modules (longer time commitment) and the CDC study arm only completed the CDC module. Time commitment is an important issue for the implementation of prevention strategies. Thus, incorporating prevention strategies into existing infrastructures must be done carefully. As noted in previous research related to other prevention programming, such as the FIFA 11+ (for lower extremity injuries), having coach and organization buy-in is integral [35]; however, this may also require more flexibility for the implementation of content [36]. However, content delivery that is variable may result in similar variability in effectiveness, with non-immersive strategies being the least effective [12].

Further, although still generally rated positively, topics, such as reducing concussion risk and keys to effective conversations (related to concussion prevention and management) and the ability to use the received information on a regular basis were rated lower than other topics as these are likely less familiar to families of youth sport participants. Previous research suggests the concerns constituents within sport settings have in regard to talking with others about concussions. For example, a study examining parents of middle school sport athletes noted that concussions were not discussed unless one had occurred to a player on the team, and that they believed that they were not the appropriate people to discuss concussions as they felt unqualified, as opposed to doctors, nurses, and other medical professionals [10]. Further, previous research has noted concerns about how data regarding concussion knowledge have been collected in study samples [37,38]. On one hand, studies have concluded that their samples demonstrated high concussion knowledge; on the other hand, other studies have countered that such findings may be a function of the measurements used that may inflate scores (e.g. symptom checklists, true-and-false prompts) [37,38]. Moreover, despite such studies noting high knowledge, cases of nondisclosure and delayed reporting are still noted in the literature [39].

The findings emphasize the need for educational efforts that highlight concussion signs and symptoms, while helping individuals consider their recognition in relevant contexts (e.g. during sports participation scenarios and non-sport settings) and feel empowered and confident in advocating for proper response and management. Further, the development of concussion prevention programming must acknowledge the difficulties in translating education into actual applied practices and behaviors. Such general concerns have been previously addressed within other concussion prevention programming [12]. Additional efforts to continue improving the delivery of programming content and how it can be applied feasibly are pertinent to ensuring their potential benefits are realized.

### Effects of training modules on concussion-related outcomes

Overall, there was insufficient evidence that receiving the TRAIN training module with the CDC training module provided any additional increases in knowledge, attitudes, intentions, and self-efficacy over the CDC training module alone. Effect sizes were generally small, potentially highlighting the need for larger samples than that of the present study to ensure sufficient statistical power. Last, proposed scale measures for knowledge, intentions, and self-efficacy had low internal consistency (Cronbach’s alpha *α* < 0.80), resulting in keeping analyses for each item discrete. Thus, continued refinement of the modules and measures of interest is warranted.

Among those identifying as female, the TRAIN + CDC study arm was found to have an increase across time in their confidence to have conversations with other adults about concussion management; no such change was found in the CDC study arm. On the other hand, only the CDC study arm was found to have an increase in their knowledge score related to symptoms being more likely to go away sooner when a youth athlete with a suspected concussion was taken out of a game/practice. Similar findings were not found among the sample identifying as male. Previous research has heavily discussed gender-based differences related to concussion outcomes. This includes differences in concussion-related knowledge and attitudes [22,40,41]. Although our study is limited in its results related to gender differences, its findings coupled with the previous research emphasize that concussion education and prevention materials need to consider the diversity of the populations that are reached. While more tailored educational provisions may increase the upfront burden for those designing and implementing these strategies, the investment may improve engagement as well as overall outcomes targeted by the intervention.

Most statistically significant findings pertained to time effects across both study arms. Participant scores increased in terms of aspects of knowledge of and confidence in identifying concussion signs and symptoms. As previously noted, presentation mode may play a role in the ability of individuals to be able to retain information gained from training. Likewise, a previous review of the concussion education literature posited that while online modules may be beneficial, in-person trainings may be more effective [42]. Although the CDC module included materials gathered from CDC resources, we ensured that it included interactive components to keep its audience engaged, which may help to explain the gains seen in the sample. Thus, it is important for interventions to consider opportunities to allow their intended audiences to be engaged; this includes working with sport organization constituent [15], providing interactive modules with simulated scenarios and decision-making [14], and formative evaluation that involves expert feedback in their development [13].

It is important to note that the data collection of ‘post’ measures occurred ∼1 week after the completion of the training modules. As a result, it is possible that more significant findings may have been found if ‘post’ data collection occurred immediately upon completion of module. We had discussed including an immediate follow-up data collection window, but opted for one week, as we were concerned about participant fatigue. A systematic review of concussion-related interventions noted that although included studies found short-term increases in knowledge, scores tended to return to baseline in the long-term [13]. Choosing to use a long-term follow-up window may result in more valid assessments of actual intervention benefits. For sport settings, this can occur naturally *via* data collection at the post-season [12,15].

### Limitations

As with all research, this study had limitations. First, this study relied on a convenience sample of parents of MS students. The characteristics of the sample (e.g. distributions of demographics, proportions having formal concussion education) may not be representative of the population of middle school parents in the US. As a result, findings may not be generalizable to the entire population, or to parents of athletes at other ages or levels of play (e.g. high school, collegiate). In addition, the study had a relatively high attrition rate. Those completing the training modules and data collection protocol may have differed from those who had dropped out. Thus, findings may not be representative of the initial full study groups. Last, given challenges related to recruitment during the COVID-19 pandemic, caution should be taken as our sample size may not have yielded sufficient statistical power; estimated *post-hoc* power for the group*time interaction ranged from ∼0.36 to 0.71 for effect sizes of 0.20 to 0.30, respectively.

Second, as a novel study utilizing this POL-informed approach, we aim to continue refining the training modules based on participant feedback. With each refinement effort, the results from the current study may not be completely applicable and may require additional evaluation. However, given previous formative work, the core components in these modules would remain.

Last, it is important to consider the limitations of our chosen measurements. It was originally intended that our collected data would yield scale measures of concussion-related knowledge, attitudes, intentions, and self-efficacy. Our measures were based on previously developed and refined measurements [24]. However, we made additions and edits to further add to our understanding of these concussion-related concepts. When evidence of low internal consistency was found (i.e. Cronbach alphas <0.80), we examined each item singularly where applicable. *A priori* decisions based on previous literature [22,27–29,32,33] were also made to use parametric methods and covariate selection and treatment. Continued research to further refine measurements, covariate selection, and analyses of these important concepts is required.

## Conclusion

There was limited evidence of increased knowledge and self-efficacy one week after completing training modules that sought to educate parents about concussion-related prevention and management and also to feel confident in discussing such prevention and management needs within their peer networks. Although there were no differences between those who received both modules and those who only received the CDC education module, both study arms saw beneficial increases in knowledge and self-efficacy. The findings, coupled with previous research, highlight the need to consider manners to continue improving the design and effectiveness of such training modules. This includes providing the user with strategies to feel confident in identifying concussion signs and symptoms and to advocate for the provision of concussion prevention and management. Such strategies should also be included in relevant contexts (e.g. common sport-related scenarios).

## Supplementary Material

Supplemental Material

## Data Availability

The data that support the findings of this study are available from the corresponding author, ZYK, upon reasonable request.
